# Mifepristone Repurposing in Treatment of High-Grade Gliomas

**DOI:** 10.3389/fonc.2021.606907

**Published:** 2021-02-18

**Authors:** Monserrat Llaguno-Munive, Maria Ines Vazquez-Lopez, Rafael Jurado, Patricia Garcia-Lopez

**Affiliations:** Laboratorio de Farmacología, Subdirección de Investigación Básica, Instituto Nacional de Cancerología, Mexico City, Mexico

**Keywords:** brain cancer, glioma, mifepristone, repurposing, repurposed drug, resistance

## Abstract

Glioma is the most common and aggressive primary tumor of the central nervous system. The standard treatment for malignant gliomas is surgery followed by chemoradiotherapy. Unfortunately, this treatment has not produced an adequate patient response, resulting in a median survival time of 12–15 months and a 5-year overall survival of <5%. Although new strategies have been sought to enhance patient response, no significant increase in the global survival of glioma patients has been achieved. The option of developing new drugs implies a long and costly process, making drug repurposing a more practical alternative for improving glioma treatment. In the last few years, researchers seeking more effective cancer therapy have pursued the possibility of using anti-hormonal agents, such as mifepristone. The latter drug, an antagonist for progesterone and glucocorticoid receptors, has several attractive features: anti-tumor activity, low cytotoxicity to healthy cells, and modulation of the chemosensitivity of several cancer cell lines *in vitro*. Hence, the addition of mifepristone to temozolomide-based glioblastoma chemotherapy may lead to a better patient response. The mechanisms by which mifepristone enhances glioma treatment are not yet known. The current review aims to discuss the potential role of mifepristone as an adjuvant drug for the treatment of high-grade gliomas.

## Introduction

Glioma is the most common primary neoplasm in the central nervous system, making up approximately 30–45% of tumors in the central nervous system (CNS). These tumors are very invasive, making a complete surgical resection impossible (1).

The World Health Organization (WHO) has classified gliomas into four grades, based on degree of malignancy. Grade 1 tumors, most frequently found in children, are considered gliomas with a low risk of malignancy and a reduced potential for proliferation. Grade 2 tumors, appearing mainly in young adults 20–30 years of age, are slow growing and in some cases have a tendency to progress to a more malignant tumor. Grade 3 tumors present a high rate of mitotic activity and are very invasive, giving a median survival time of 1–3 years. Grade 4 tumors, also called glioblastoma multiforme (GBM), correspond to the maximum degree of malignancy, being characterized by rapid growth, necrotic zones, and an accelerated rate of progression. Grade 3 and 4 are malignant or high grade gliomas, mainly characterized by a poor prognosis, resistance to chemoradiotherapy, and rapid tumor recurrence (2).

Additionally, in 2016, the WHO reclassified the gliomas by molecular profiling. This classification includes mutation in isocitrate dehydrogenase 1 or 2 (IDH1 or IDH2), wild type IDH, or not otherwise specified (NOS). The IDH proteins are involved in the conversion of isocitrate into alpha-ketoglutarate in the tricarboxylic acid cycle. In low-grade gliomas, isocitrate dehydrogenase (IDH) mutations were found with higher frequencies (83%), compared to grade III astrocytoma (70%) or primary and recurrent GBM (5%) (3). These mutations have been correlated with better prognosis, leading to a higher median survival in patients with IDH mutations in all gliomas.

Malignant gliomas are rare tumors in epidemiology. They constitute 2% of the total cases of cancer in women and 2.8% for men (4). In spite of the low prevalence of gliomas, it is urgent to find an effective medical treatment because the average survival rate is under 2 years.

### Physiopathology of Glioma Development

Diverse reports in “The Cancer Genome Atlas” (TCGA) describe three main signaling pathways involved in the pathogenesis of gliomas. This include: RTK/RAS/PI3K (receptor tyrosine kinase, RAS, phosphatidylinositol 3-kinase), p53, and retinoblastoma (5). Another important pathway involved in glioma pathology is that related to angiogenesis (the formation of new blood vessels from a pre-existing vascular network). One of the main stimulants for the expression of angiogenic factors is hypoxia. This factor induces the synthesis of vascular endothelial growth factor (VEGF), which is considered the most important signal protein mediating angiogenesis.

Various strategies have been utilized to inhibit the expression of VEGF, such as the bevacizumab, a humanized monoclonal antibody. According to two prospective phase-III trials, the addition of bevacizumad to the first-line treatment (temozolomide and radiotherapy) did not improve overall survival in patients with newly diagnosed glioblastoma. Progression-free survival was prolonged but did not reach the pre-specified improvement target (6, 7).

Yang et al. evaluated the transcriptome of patients with glioblastoma, observing differences between men and women in the gene pathways associated with survival. In men, the pathways most commonly linked to overall survival participate in cell division. Thus, the pharmacological blockage of the progression of the cell cycle may be more effective in men. In women, the genes most closely related to overall survival are involved in the regulation of invasiveness; therefore, drugs targeting integrins could function better in women (8).

Another common focus of research on glioma tumors is the PI3K/Akt signaling pathway (9). The *PTEN* gene, encoding for a protein that inhibits the PI3K/Akt pathway, is inactivated in 40 to 50% of the cases of glioblastoma. This pathway is closely related to resistance to treatment because its activation promotes proliferation, invasion, and angiogenesis, and has been related to the conversion of anaplastic astrocytoma (grade 3) into GBM (grade 4) (9, 10).

### Resistance to Treatment

Treatment of malignant gliomas begins with surgical resection, which is often incomplete, leaving residual cells that are capable of migrating and proliferating. Therefore, surgery is accompanied by 60 Grays (Gy) of radiotherapy (2 Gy/daily) and chemotherapy with the alkylating agent temozolomide (75 mg/m^2^, daily by 6 weeks), followed by a dose of maintenance of 150–200 mg/m^2^ for 5 days every 28-day cycle for six cycles. However, it has not given the desired response, resulting in a median survival time of 15 months (11, 12).

Among the most important challenges in the treatment of gliomas are the restrictions on access to the brain imposed by the blood-brain barrier (BBB), the limited response to therapy, and neurotoxicity stemming from the treatments (13). The vast majority of chemotherapy treatments for glioma tumors have failed due to the reduced concentrations of the drug that reach the CNS (14, 15).

The mechanism of action of temozolomide consists of inhibiting the replication of DNA. This prodrug is spontaneously transformed into monomethyl triazeno imidazole carboxamide (MTIC) upon entering the organism. The cytotoxicity of MTIC is reportedly caused by the alkylation of guanine at positions O6 and N7 of DNA (16). Unfortunately, the adducts generated are removed by the repair enzyme 06-methylguanine-methyltransferase (MGMT), leading to resistance and recurrence. The silencing of the MGMT gene seems to improve the response to treatment in patients receiving alkalizing agents (17). The methylation of the CpG island of the MGMT enzyme promoter is associated with better survival of patients with high grade gliomas that are given alkylating agents as chemotherapy.

**Figure 1:**
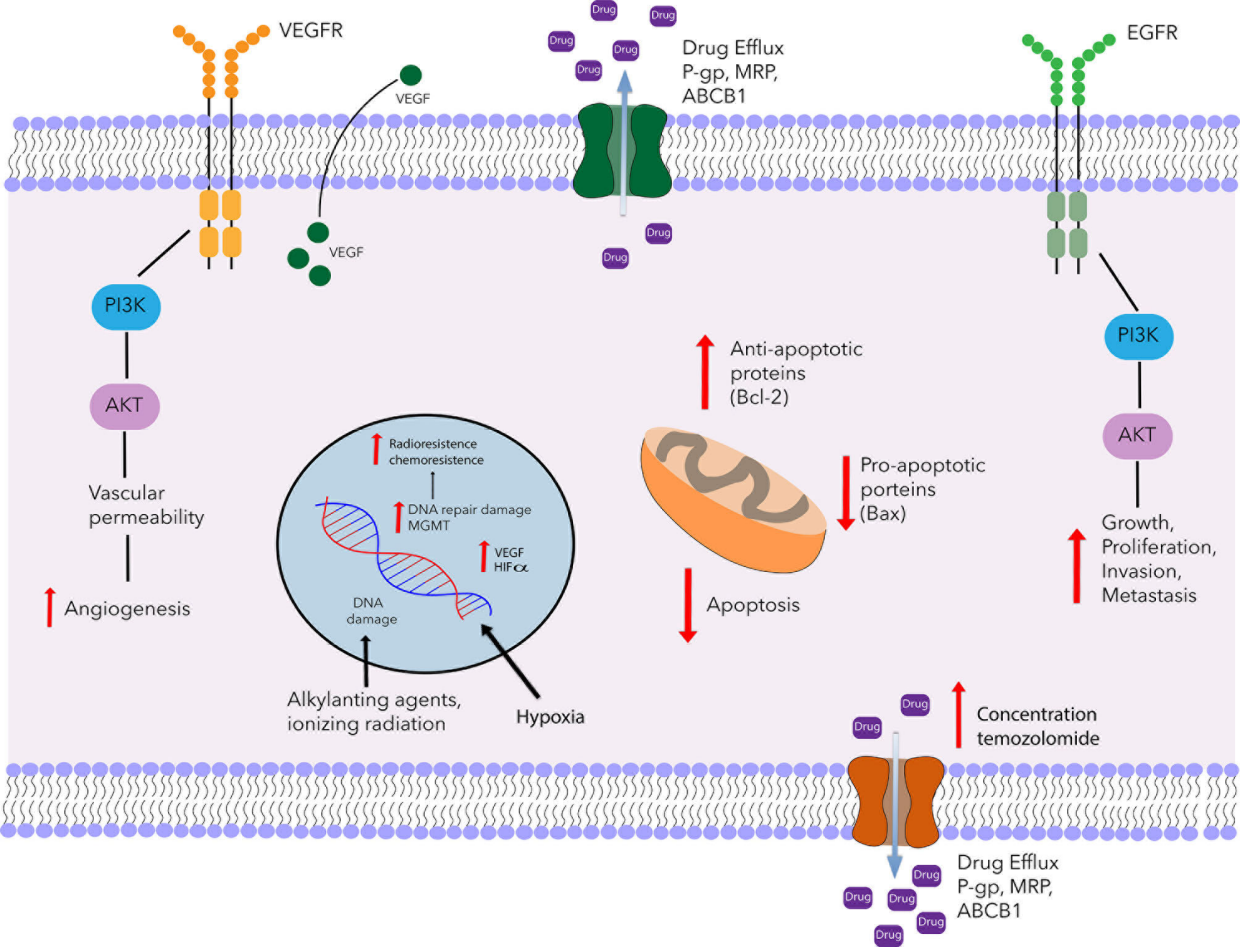
Schematic portrayal of resistance mechanisms of glioblastoma. Glioblastoma multiforme is characterized by an angiogenic tumor and resistant to chemotherapy. One of the mechanisms of temozolomide resistance is mediated by MGMT, which removes methyl group O6-MeG lesión and restores the cellular replication of glioblastoma cells. Another mechanism of resistance is the ATP-dependent transporters family (P-gp, MRP, BCRP). These proteins are involved in the uptake and efflux of several drugs including temozolomide. Also, in glioblastoma tumors, there is an increase of VEGF expression and over-expression of VEGFR promoting the formation of tumor blood vessels. Another tyrosine kinase receptor that is involved in chemoresistance is the epidermal growth factor receptor (EGFR), activates MAPK, and PI3K signaling promoting growth, proliferation, invasion, and metastasis. On the other hand, it has been reported that radioresistance is partly due to the presence of hypoxic regions, hypoxia is associated with tumor angiogenesis and invasiveness, therapeutic resistance, and poor prognosis. In addition to the resistance mechanisms described above, there is dysregulation of the apoptosis genes, such as the up-regulation of Bcl-2 and the down-regulation of Bax. The combination of these mechanisms contributes to a chemo-radioresistance.

Besides the repair mechanisms for damaged DNA, another mechanism involved in resistance to treatment is an insufficient accumulation of the drug at the target site stemming from alterations in transporters dependent on ATP (**Figure 1**) (18). Among such transporters overexpressed in the BBB and glioma tumor cells are the P-glycoprotein multidrug resistance protein 1 (P-gp/MDR1/ABCB1) and the multidrug resistance-associated protein (MRP/ABCC1) (19). Several drugs showing affinity for P-gp include actinomycin D, taxanes, anthracyclines, and temozolomide (20, 21).

The blockage of the ATP-dependent transporters could lead to a considerable enhancement of glioma treatment. Temozolomide is reported to compete with substrates of P-gp, thus blocking their activity (20), and to diminish the activity of P-gp by inhibiting its ATPase (22). However, there is controversy about whether temozolomide actually increases or decreases the expression of this protein. According to one study, temozolomide boosts the expression of P-gp through the EGFR pathway (23), while another found a temozolomide-induced reduction in the expression of P-gp in BBB cells caused by the methylation of the WNt3a gene promoter (24). A reduced expression of P-gp would sensitize glioma cells to treatment.

### Drug Repurposing for Combination With Temozolomide in the Treatment of Gliomas

Despite extensive research on the design and development of new molecules to achieve a better response to glioma treatment, patient overall survival, and the median survival time have not yet improved. One strategy employed in recent years is the repositioning of drugs, which consists of finding new applications for approved drugs. An advantage of drug repurposing is that is known a profile of safety and efficiency, making it a candidate for rapid incorporation into other clinical treatments, implying less risk and greater cost-benefit.

Cost is an important factor in the development of new compounds for the treatment of diseases. The cost of developing new drugs is generally in the range of a billion dollars (25, 26). The US Food and Drug Administration (FDA) considers glioblastoma as a rare disease, which limits the initiative of the pharmaceutical industry to invest in the development of new drugs for this neoplasm. Hence, drug repurposing may be the most suitable strategy under these circumstances. The administration of multiple therapeutic agents with various targets in malignant gliomas likely provides more benefits than standard chemotherapy based on a single agent.

Although intense research efforts have been made to improve the current treatment of glioblastoma, there are scant reports on the repositioning of drugs for use in combination with temozolomide. An experimental and clinical study on human glioma cells, on an animal model, and in patients with recurrent GBM demonstrated that the combination of temozolomide with inhibitors of the tumor promoter gene *GSK3β* (glycogen synthase kinase-3 β) reduces in the progression of the disease and protects against the neurodegenerative effects provoked by radiotherapy (27). Such inhibitors include cimetidine, valproate, olanzapine, and lithium carbonate (commonly prescribed to treat gastro-duodenal ulcer, epilepsy, schizophrenia, and bipolar disorder).

Temozolomide was tested in combination with hydroxyurea on an orthotopic experimental model of glioma, finding an increase in the percent survival of the animals (28). Roix *et al.* (2014) evaluated 182 compounds *in vivo*, identifying three (candesartan, risedronate, terbinafine) that lead to a better response of animals when given in combination with temozolomide. Preclinical trials are still necessary to explore their efficacy (29).

In the last few years, our group has investigated mifepristone (an abortifacient drug) as a plausible repurposing candidate for treating a wide range of cancers. A synergistic action has been demonstrated when it is combined with cisplatin or temozolomide plus radiation (30–34).

### The Repositioning of Mifepristone for Cancer Treatment

Mifepristone (RU486) was the first antiprogestogen developed. In 1981, it was described as an antagonist of glucocorticoid, progesterone, and androgen receptors. Its first use was as an emergency contraceptive pill to induce abortion (35). In the year 2000, mifepristona was approved by the FDA as an abortive agent. In practice, this drug has been utilized for a great variety of diseases and clinical conditions, including the termination of pregnancy, endometriosis, Cushing’s syndrome, and metabolic syndrome (36).

Due to its antiproliferative and antimetastatic activity, mifepristone has been evaluated individually as a potent agent in metastatic ovarian cancer with positive progesterone receptor (PR) (37). However, the loss of PR may represent a more aggressive panorama in the development and progression of cancer. Mifepristone is also known to produce effects not mediated by hormonal receptors, acting on hormone receptor negative cells. It was found to inhibit cell growth in ten different PR negative cancer cell lines. The mechanism described was a decrease in the activity of checkpoint Cdk2 of the cell cycle, generating cell cycle arrest in phase G1 (38). Hence, the effect of mifepristone *does not require* of the presence of the PR. Additional studies carried out on PR negative cancer cells support the finding of mifepristone-induced antiproliferative effects on breast (39), cervical (40), endometrial (41), ovarian (42) and prostate cancer (43).

Nowadays, there are several clinical trials in which mifepristone is used alone or in combination with another drug to treat different types of cancer. In the case of prostate cancer, a phase II clinical trial has been conducted. In this study, 200 mg of mifepristone was administered daily, which was well tolerated, with no incidence of clinical adrenal insufficiency (44).

Another clinical trial (phase I/II) is currently recruiting patients with metastatic, castration-resistant prostate cancer. The first goal is to establish the safe and pharmacologically active dose of mifepristone and enzalutamide (androgen receptor antagonist). The second goal is to determine if mifepristone in combination with enzalutamide delays time to prostate-specific antigen (PSA) progression compared to enzalutamide alone [ClinicalTrials.gov identifier (NCT number): NCT02012296].

On breast cancer patients, the combination of paclitaxel-charged nanoparticles plus mifepristone (300 mg) was evaluated in a phase I trial. This combination was found to be well tolerable by patients (45).

Mifepristone administration has been documented to improve both length and quality of life in patients with advanced non-small cell lung cancer (46, 47) and renal cancer (48). In advanced pancreatic cancer this drug had palliative benefits (49). Mifepristone seems to be well tolerated at a dose of 200–300 mg.

### Mifepristone as a Sensitizing Agent for Malignant Gliomas

Epidemiological studies show that the incidence of glioblastoma is approximately 40% higher in men than in woman. Furthermore, the woman/men ratio is lower in pre-menopausal women, and increases in parallel with the decrease in female hormone levels, which suggests that these hormones have preventive effects on gliomagenesis (50). These results correlate with a lower incidence and better overall survival for women with brain tumors (50, 51). Estrogens also improved the percentage of animal survival in an orthotopic model of experimental glioma (52). These data suggested that estrogen might be responsible for better overall survival.

On the other hand, it has been found that the expression of glucocorticoid and progesterone receptors is elevated in patients with high-grade glioma. It is known that this type of receptors play an important role in cell proliferation. In this way progesterone and glucocorticoids could promote the development of gliomas.

Experimental studies have showed that progesterone is capable of stimulating the infiltration and migration of astrocyte in the rat cortex (53). Mifepristone, due to its anti-progestational and anti-glucocorticoid action, blocks the capacity of progesterone to stimulate the growth, migration and invasion of human astrocytoma cells lines (54, 55). Nowadays, several clinical and preclinical studies are being carried out to understand and confirm the impact of steroid hormones on gliomatosis.

Recently it was demonstrated the participation of progesterone in the growth of glioblastoma stem cells (56). Several studies have suggested that stem cells may be responsible for resistance and recurrence in glioblastoma. It is still unknown whether mifepristone could inhibit the growth of glioma stem cells; however, some authors have shown that mifepristone reduces the breast cancer stem cells population (39, 57). Therefore, future research is required to determined whether mifepristone could regulated glioma stem cells.

The methylation of the MGMT promoter is a prognostic factor associated with increased temozolomide response and overall survival in glioblastoma. Schiffgens *et al.* (2016), suggested that the silencing of the MGMT gene may be influenced by the sex. In this study a greater proportion of females presented promoter methylation in comparison with males. However, the authors mentioned that it is necessary to corroborate these results due to the low sample size in its study (50, 58).

Glucocorticoid receptor is expressed in neurons, oligodendrocytes, astrocytes, and microglia of the brain (59). Glucocorticoids as dexamethasone are frequently used in patients with high-grade glioma as a therapy to address edema and increased intracranial pressure. However, its use is controversial on glioblastoma progression (60). An increase in proliferation, angiogenesis, invasion and anti-apoptotic effects has been described in preclinical studies. In addition, dexamethasone seems to decrease the efficacy of temozolamide (61, 62). Therefore, the addition of mifepristone, as GR antagonist, could increase the effect of temozolomide and decrease cellular proliferation (60).

According to some authors, the glucocorticoids are involved in eliciting the expression of the MGMT gene, which means that these drugs could contribute to an elevated MGMT protein level. Biswas *et al*. and Ueda *et al*. detected an upregulation of MGMT in glioblastoma cell lines during glucocorticoid treatment (63, 64).

Mifepristone seems to have great potential for glioma treatment as a glucocorticoid receptor antagonist. In C6 glioma cells implanted intracranially in rats, our group recently described the capacity of mifepristone to reduce the expression of the MGMT protein. This led to a higher rate of apoptosis and consequently to diminish tumor proliferation in rats treated with the mifepristone/temozolomide combination (33, 34). Consequently, one of the mechanisms possibly involved in the mifepristone-induced sensitization to temozolomide is the modulation of the expression of the MGMT enzyme.

It is still unknown whether mifepristone epigenetically inhibits MGMT. However, different nuclear transcription factors as SP1, AP-1, NF-kappa B, and HIF-1alpha, can activate the transcription of MGMT gene in glioblastoma (65). Some of the above-mentioned transcription factors may participate in MGMT gene regulation by mifepristone.

Mifepristone has also been found to inhibit the expression of the VEGF, which is overexpressed in glioblastoma. We find that there was an additive effect by temozolamide and mifepristone in the inhibition of VEGF levels. Mifepristone also blocks the function of drug efflux proteins such as P-gp. This protein is highly expressed by endothelial cells in gliomas, and a key role has been attributed to P-gp in the chemoresistance of gliomas. In our work we show the participation of mifepristone in the inhibition of P-gp expression, and on the increased intracerebral concentration of temozolomide. The tumor growth rate was slower than that found with temozolomide alone, indicating a chemo-sensitizing effect. According to the current results, mifepristone could contribute to the modulation of tumor relapse in glioblastoma by decreasing the levels of VEGF, MGMT, and P-gp (34). Further research is needed to explore other mechanisms of drug resistance of glioblastoma tumors.

Several clinical studies show that mifepristone is capable of crossing the BBB and has demonstrated palliative effects on brain tumors, such as meningiomas (66, 67) and glioblastoma (68). It has also been found to improve the quality of life of patients with glioma. These characteristics make mifepristone an attractive repurposing candidate for the treatment of malignant gliomas. It is considered a safe drug, which even with prolonged use has relatively mild adverse effects, such as fatigue, nausea, vomiting and diarrhea. The administration of mifepristone monotherapy was reported for two clinical cases of cancer: a patient with non-small-cell lung carcinoma (NSCLC) and brain metastasis, and another with bilateral kidney cancer and metastasis. In both patients, the result was a better quality of life.

Based on the experimental data, as well as in clinical trials, mifepristone appears to be a promising approach against glioblastoma. The addition of mifepristone to glioblastoma treatment could improve the quality of life of patients, and has the potential to control the progression of tumors.

### Future Directions

The continuous effort to identify new treatments for glioblastoma is yet to lead to significant improvements in the survival rate. The molecular complexity of glioblastoma has forced the scientific community to pay attention in new strategies, such as drug repurposing.

**Figure 2:**
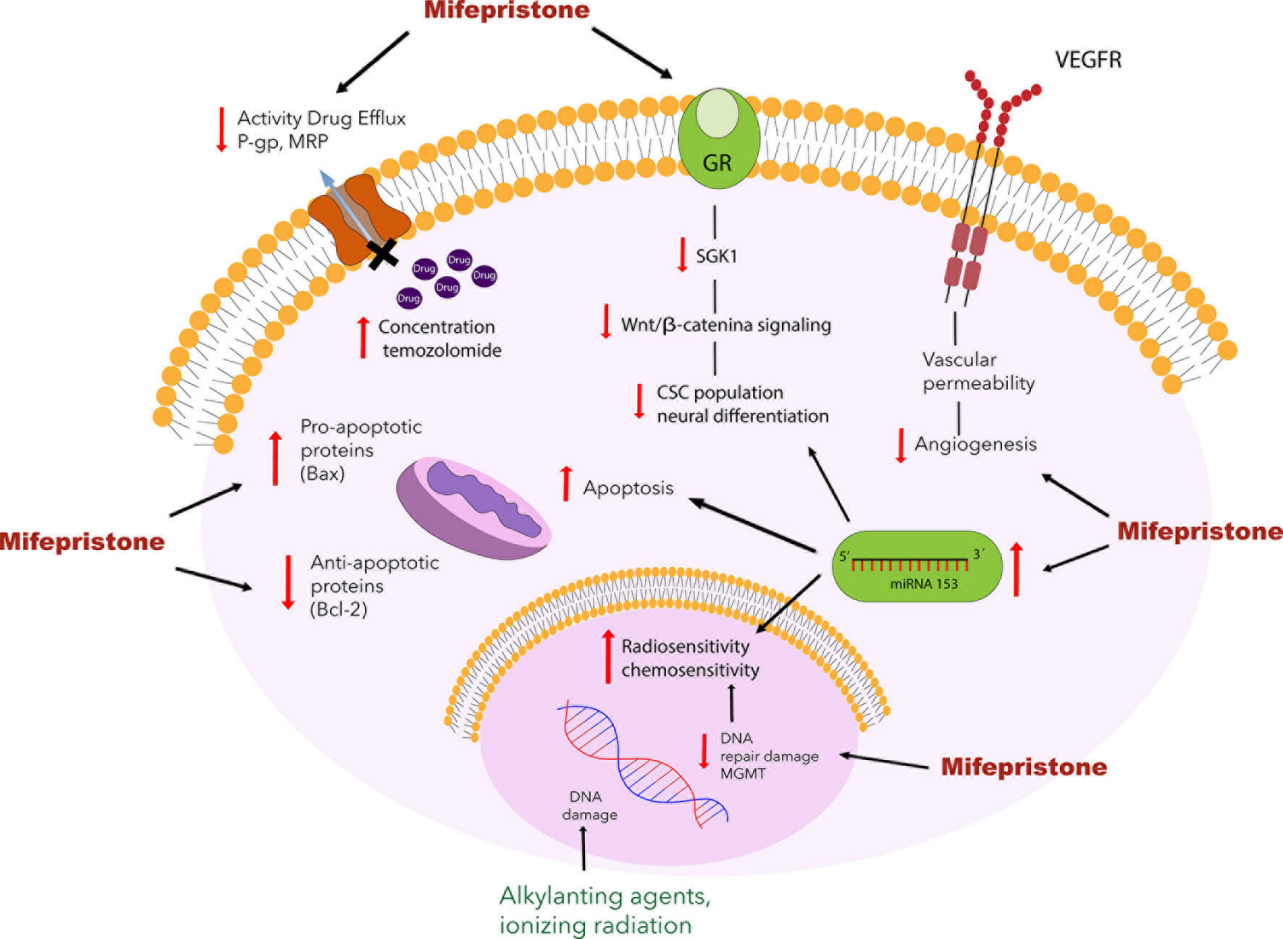
Schematic portrayal of the possible mechanisms of mifepristone as a chemo-sensitizing agent for glioblastoma. Mifepristone decrease the level of anti-apoptotic protein Bcl-2 and increase the levels of pro-apoptotic protein Bax leading to apoptosis. Mifepristone has also been found to inhibit the expression of VEGF and block the function of drug transporting proteins such as P-gp and MRP. Another mechanism involved in the mifepristone-induced sensitization to temozolomide is the modulation of the expression of the MGMT enzyme, it has been reported that mifepristone reduces the expression of the MGMT protein in combination with temozolomide, leading to apoptosis. As described elsewhere, mifepristone decreases the growth of stem cells by inhibiting the Wnt/β-catenin signaling pathway, which involves glucocorticoid receptors and increasing the miRNA-153.

Recently, new evidence has emerged about the role of stem cells in the development of cancer and resistance to therapy. Several studies have suggested that cancer stem cells could be responsible not only for reduced treatment efficacy but also their recurrence (69). One of the most challenging aspects when treating gliomas is the complete elimination of cancer stem cells. Glioblastoma stem cells are reported to abundantly express the MGMT and P-gp proteins, leading to greater resistance to treatment, a higher level of hypoxia, and more frequent tumor recurrence (70, 71). As a result, these proteins have become an important therapeutic target to improve patient response to glioblastoma treatment. As we have mentioned, several studies reported that mifepristone decreased MGMT and P-gp expression (33, 34) (**Figure 2**). Future research is required to determine whether mifepristone can regulate glioma stem cells, offer greater benefits during tumor recurrence and improve the prognosis of patients with glioma.

## Conclusion

A great variety of strategies exist for the development of new glioblastoma treatments. Yet in the vast majority of cases, none of them has yet been able to control the progression of tumors or recurrence. Mifepristone has been found to improve the quality of life of patients with glioma. It is considered a safe drug that, even with prolonged use, has relatively mild adverse effects. Considering the chemoresistance mechanisms reviewed, mifepristone could possibly be a sensitizing agent in therapy against malignant gliomas and we recommend it for clinical trials on a combined mifepristone/temozolomide plus radiation treatment for glioblastoma, which holds promise for improved therapeutic efficiency and patient overall survival.

## Author Contributions

ML-M and PG-L organized topics and contributed in manuscript drafting. MV-L and RJ collected the literature. All authors contributed to the article and approved the submitted version.

## Funding

This work was partially financed by CONACYT (Mexico) through grant CB-258823.

## Conflict of Interest

The authors declare that the research was conducted in the absence of any commercial or financial relationships that could be construed as a potential conflict of interest.
